# Transcriptome Reveals the Molecular Mechanism of the *ScALDH21* Gene from the Desert Moss *Syntrichia caninervis* Conferring Resistance to Salt Stress in Cotton

**DOI:** 10.3390/ijms24065822

**Published:** 2023-03-18

**Authors:** Honglan Yang, Qilin Yang, Dawei Zhang, Jiancheng Wang, Ting Cao, Tohir A. Bozorov, Lihua Cheng, Daoyuan Zhang

**Affiliations:** 1 State Key Laboratory of Desert and Oasis Ecology, Key Laboratory of Ecological Safety and Sustainable Development in Arid Lands, Xinjiang Institute of Ecology and Geography, Chinese Academy of Sciences, Urumqi 830011, China; 2 Xinjiang Key Laboratory of Conservation and Utilization of Plant Gene Resources, Xinjiang Institute of Ecology and Geography, Chinese Academy of Sciences, Urumqi 830011, China; 3 University of Chinese Academy of Sciences, Beijing 100049, China; 4 Research Institute of Economic Crops, Xinjiang Academy of Agricultural Sciences, Urumqi 830091, China; 5 Turpan Eremophytes Botanical Garden, Chinese Academy of Sciences, Turpan 838008, China; 6 Institute of Genetics and Plants Experimental Biology, Uzbek Academy of Sciences, Yukori-Yuz, 111226, Kibray District, Uzbekistan; 7 College of Agronomy, Xinjiang Agricultural University, Urumqi 830052, China

**Keywords:** cotton, salt tolerance, *Syntrichia caninervis*, transcriptome, transgenic technology

## Abstract

The desert moss *Syntrichia caninervis* has proven to be an excellent plant material for mining resistance genes. The *aldehyde dehydrogenase 21* (*ScALDH21)* gene from *S. caninervis* has been shown to confer tolerance to salt and drought, but it is unclear how the transgene *ScALDH21* regulates tolerance to abiotic stresses in cotton. In the present work, we studied the physiological and transcriptome analyses of non-transgenic (NT) and transgenic *ScALDH21* cotton (L96) at 0 day, 2 days, and 5 days after salt stress. Through intergroup comparisons and a weighted correlation network analysis (WGCNA), we found that there were significant differences between NT and L96 cotton in the plant hormone, Ca^2+^, and mitogen-activated protein kinase (MAPK) signaling pathways as well as for photosynthesis and carbohydrate metabolism. Overexpression of *ScALDH21* significantly increased the expression of stress-related genes in L96 compared to NT cotton under both normal growth and salt stress conditions. These data suggest that the *ScALDH21* transgene can scavenge more reactive oxygen species (ROS) in vivo relative to NT cotton and improve cotton resistance to salt stress by increasing the expression of stress-responsive genes, responding quickly to stress stimuli, enhancing photosynthesis and improving carbohydrate metabolism. Therefore, *ScALDH21* is a promising candidate gene to improve resistance to salt stress, and the application of this gene in cotton provides new insights into molecular plant breeding.

## 1. Introduction

Abiotic stresses have significant negative impacts on organisms in the environment [1,2,3]. For example, drought, salinity, heat, flooding, and other extreme environments, as well as air pollution and climate change, are the main causes of reducing plant growth and yield [3]. Soil salinity is a serious problem worldwide that often causes yield reductions in agricultural crops via severe cell dehydration and ion toxicity [4,5]. Hassani et al. suggested that soil salinity will increase more extensively due to the impacts of climate change. [6]. Therefore, it is a challenge to improve plant productivity via developing salt-tolerant cultivars, which is one of the important strategies to mitigate salt stress.

Plants produce most reactive oxygen species (ROS) in response to adverse stresses [7,8,9]. Excessive ROS can cause oxidative damage to macromolecules such as membrane lipids, proteins, and DNA and can disrupt the normal functions of plant cells, harming plant growth and reducing crop yields [10,11]. At the same time, plants in adverse conditions produce large amounts of aldehydes, which can cause metabolic disorders and lipid peroxidation in plants [12,13]. Aldehyde dehydrogenase (ALDH) is an aldehyde scavenger during the process of lipid peroxidation, which can oxidize various aliphatic and aromatic aldehydes to their corresponding carboxylic acids, scavenge ROS and prevent lipid membrane peroxidation by degrading harmful aldehydes to ensure normal physiological functions [14,15,16]. Therefore, ALDH has been of great interest in the study of the mechanism of ROS production and scavenging in plant adversity.

Cotton (*Gossypium* sp.) is the most important fiber and oilseed crop [17]. However, water constraints and salinization of cotton growing areas are challenges for fiber production [18]. High salinity can severely limit cotton growth and yield by inhibiting leaf unfolding, reducing chlorophyll content, decreasing fiber quality, reducing plant height, and increasing hyperionic and hyperosmotic stresses [4,19,20].

The identification of salt tolerance genes is an important component of salt-tolerant crops breeding through genetic engineering. Although many genes controlling high salinity response to high salinity have been identified in model plants, only a few of them have been practically applied in agricultural production.

*Syntrichia caninervis* (Mitten) Brotherus is a desiccation-tolerant model species of desert mosses and is the dominant species in the moss biological crust in Gurbantunggut desert in Xinjiang [21,22]. Therefore, *S. caninervis* is rich in stress tolerance genes. The genome of *S. caninervis* has been reported in previous studies [23], and various genetic resources have been mined and studied [24,25,26,27,28,29]. In a previous study, the *aldehyde dehydrogenase 21* gene *(ALDH21)*, which is unique to the moss, was cloned from this species, and the *ScALDH21* gene was shown to provide high drought and salt tolerance in transformed tobacco and cotton [30,31,32,33,34,35]. However, the molecular mechanism of this transgene function in assisting salt tolerance in transformed cotton has not been reported.

In this study, we analyzed the physiological responses of transgenic cotton compared to the non-transgenic plant under salt stress, and these data were combined with a comparative transcriptomic analysis. The combined analysis discovered the molecular mechanism of salt tolerance in cotton overexpressing *ScALDH21* gene. The research explains the reason for salt tolerance in transgenic *ScALDH21* cotton on the physiological and transcriptional levels. It provides the salt tolerant cotton germplasm. At the same time, the research offers salt-tolerant gene resources via a transcriptome analysis, which provides important information to better understand salt tolerance and promote molecular plant breeding.

## 2. Results

### 2.1. The Physiological Responses of ScALDH21 Cotton to Salt Stress

**Figure 1:**
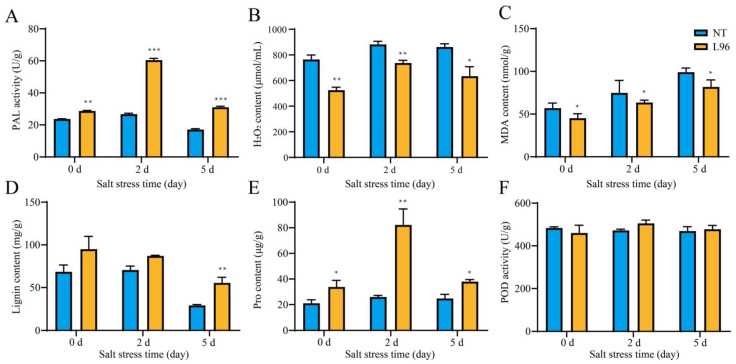
Physiological indices of NT and L96 plant under salt stress. Changes in the contents of (**A**) PAL, (**B**) H_2_O_2_, (**C**) MDA, (**D**) lignin, (**E**) Pro, and (**F**) POD in NT and L96 under salt stress (150 mM NaCl). Data represent the mean ± SD from three biological replicates. Significance was determined by the least significant difference, and asterisks indicate statistically significant differences from NT (* *p* < 0.05, ** *p* < 0.01, *** *p* < 0.001).

Previous studies confirmed that the survival rate of transgenic *ScALDH21* cotton (L96) increased under salt stress conditions, and several physiological parameters increased, such as the chlorophyll content, net photosynthetic rate, stomatal conductance, transpiration rate, and instantaneous water use efficiency, compared to those of NT cotton at the seedling stage [36]. To better understand the physiological changes of NT and L96 cotton under salt stress, we measured the biochemical contents of phenylalanine ammonia lyase (PAL), hydrogen peroxide (H_2_O_2_), malondialdehyde (MDA), lignin, proline (Pro), and peroxidase (POD) at different time points after salt stress treatment (Figure 1). The proline content and PAL activity demonstrated similar accumulation patterns at 0, 2, and 5 days after treatment that were significantly higher compared to NT cotton. The highest accumulation of these parameters for L96 line was observed 2 days after salt treatment, whereas the levels of these parameters at this time point were unchanged in NT cotton compared to 0 day. The lignin content was increased in the L96 cotton at all time points, but a significant increase was observed at 5 days after salt treatment. The hydrogen peroxide and MDA contents were decreased in the L96 line compared to NT cotton at all time points. No significant differences in the POD level were observed between the NT and L96 plants or between the time points (Figure 1).

### 2.2. Analysis of Differentially Expressed Genes during Salt Stress

**Figure 2:**
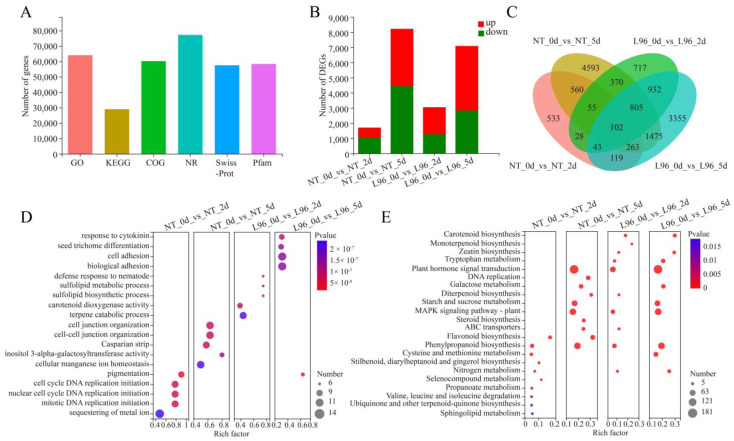
Transcriptome annotation and functional enrichment of DEGs. (**A**) Functional annotation of genes in GO, KEGG, COG, NR, Swiss-Prot, and Pfam databases. (**B**) Differential expression analysis of samples under salt stress. (**C**) Venn analysis of DEGs. (**D**) GO enrichment analysis of DEGs. (**E**) KEGG enrichment analysis of DEGs.

To understand the role of transgene in the molecular mechanism of salt stress tolerance of L96 cotton, we sequenced and analyzed the transcriptome of NT and L96 cotton at different time points after salt stress (Figure S1). The sequence analysis showed that 178,243 expressed transcripts were detected, including 98,198 known transcripts and 80,045 novel transcripts. A blast analysis of these transcripts grouped them into 68,403 known genes and 11,350 novel genes. A functional database annotation analysis of the expressed transcripts showed that 64,059 genes were annotated in the gene ontology (GO) analysis, 28,990 genes were annotated in the KEGG analysis, and large numbers of genes were annotated in the COG, NR, Swiss-Prot, and Pfam databases (Figure 2A). To better understand the salt tolerance mechanism, we compared NT and L96 salt stress samples at different time points. The number of differentially expressed genes (DEGs) increased with the duration of salt stress for both genotypes. There were 1703 DEGs in the NT_0d_vs_NT_2d group, with 659 upregulated genes and 1044 downregulated genes, and in the NT_0d_vs_NT_5d group, there were 8223 DEGs, with 3786 upregulated genes and 4437 downregulated genes. The L96_0d_vs_L96_2d group had 3052 DEGs, with 1789 upregulated genes and 1263 downregulated genes, and there were 7094 DEGs in the L96_0d_vs_L96_5d group, with 4259 upregulated genes and 2835 downregulated genes (Figure 2B). Analysis of the Venn diagram showed that a total of 102 overlapping DEGs were found among the four different groups (Figure 2C). A GO enrichment analysis of the different groups showed that NT cotton was mainly enriched for DNA replication initiation (cell cycle DNA replication initiation, and nuclear and mitotic cycle DNA replication initiation) after 2 days of salt stress, while L96 cotton was mainly enriched for the terpene catabolic process and carotenoid dioxygenase activity. NT cotton was mainly enriched for cellular manganese ion homeostasis and cell junction organization after 5 days of salt stress, while L96 cotton was mainly enriched for biological adhesion and the response to cytokinin (Figure 2D). A KEGG enrichment analysis of the different groups showed that NT and L96 cotton had similar response patterns during salt stress, where plant hormone signal transduction and the MAPK (mitogen-activated protein kinase) signaling pathway were significantly enriched, and the number of related enriched genes increased with an increase in the salt stress time. L96 cotton was also enriched for plant hormone signal transduction and the MAPK signaling pathway after 2 days of salt stress, but this was not observed in NT cotton (Figure 2E). We conducted quantitative PCR (qPCR) to validate the transcriptome data. The changes in the selected genes showed similar trends between the transcriptome and the real-time fluorescence quantitative PCR (Figure S2).

### 2.3. Comparison of Differentially Expressed Genes under Salt Stress Conditions

In order to determine the differences in the transcriptome between TheL96 and NT cotton plants under salt stress, the transcriptome data were compared at different time points. Under the normal growth condition (day 0), there were 3007 DEGs in NT_0d_vs_L96_0d, of which 1480 were upregulated and 1527 were downregulated. There were 2488 DEGs in NT_2d_vs_L96_2d, of which 1314 were upregulated and 1174 were downregulated at 2 days post-treatment. A total of 7288 DEGs were detected in NT_5d_vs_L96_5d, of which 4545 were upregulated and 2743 were downregulated (Figure S3A). NT and L96 cotton had 300 DEGs co-selected under salt stress treatments at different time periods (Figure S3B). A GO enrichment analysis of the DEGs in the different groups showed that in NT_0d_vs_L96_0d DEGs were mainly enriched in the extracellular region, the mitochondrial cell cycle process and apoplast, and others. In NT_2d_vs_L96_2d and NT_5d_vs_L96_5d, DEGs were mainly enriched in plant-type primary cell wall biogenesis, trichome biogenesis and cell junction organization (wall biogenesis, trichome differentiation, and biological adhesion), and others (Figure S3C). A KEGG enrichment analysis of the different groupings of DEGs showed that in NT_0d_vs_L96_0d DEGs were mainly enriched in plant hormone signal transduction, the MAPK signaling pathway, and plant-pathogen interaction, and in NT_2d_vs_L96_2d, DEGs were mainly enriched in starch and sucrose metabolism and flavonoid biosynthesis. DEGs in NT_5d_vs_L96_5d were mainly enriched in plant hormone signal transduction, phenylpropanoid biosynthesis, starch and sucrose metabolism, and flavonoid biosynthesis (Figure S3D).

### 2.4. Weighted Correlation Network Analysis Mines Key Pathways in Transgenic Cotton

**Figure 3:**
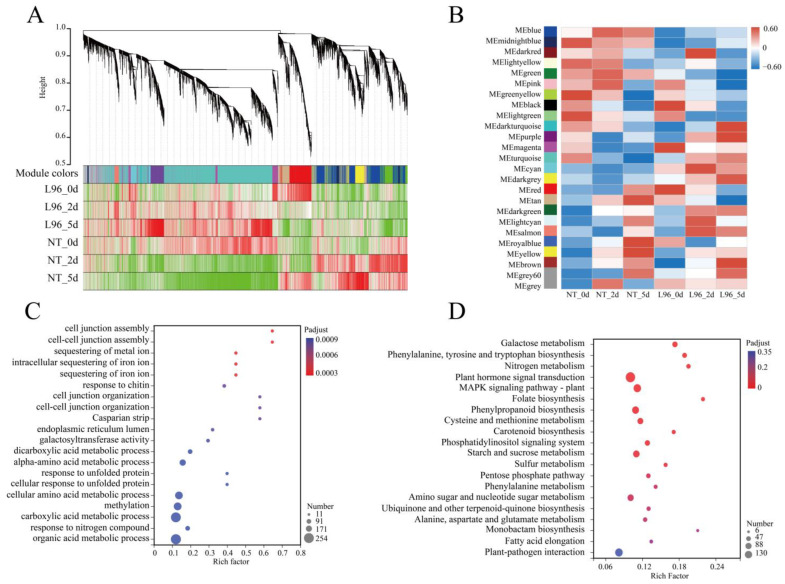
WGCNA analysis of specifically expressed genes in L96. (**A**) Co-expression network modules are enriched. Each module is given a different color. (**B**) Module–sample relationships. Each row corresponds to a color module and each column corresponds to a sample. (**C**) GO enrichment of L96-specific expressed genes. (**D**) KEGG enrichment of L96-specific expressed genes.

In order to discover the most critical signaling pathways from the many differential genes, we performed a weighted correlation network analysis (WGCNA) on NT and L96 cotton under salt stress. First, a principal component analysis and clustering were performed for the different groups, and the results showed that there were significant differences between the different lines and salt treatment times. A dispersed sample in NT_2d samples was removed from the clusters to make the subsequent analysis accurate (Figure S4A,B). Second, all genes were preprocessed for clustering classification, and 25,063 genes were divided into 25 modules, among which the maximum number of genes in the turquoise module was 6879, and a correlation analysis was performed for the different modules (Figure S4C,D). Based on the correlation analysis between the modules and salt stress treatments, we screened out the modules (MEred, MEmagenta, MEblack, MEdarkred, MEcyan, MEdarkturquoise, MEpurple, and MEbrown) that were significantly correlated (*p* < 0.01) in L96 cotton, from which the genes in these modules were selected out for subsequent analysis (Figure 3A,B). The genes in the modules from the WGCNA that were significantly associated with L96 cotton were enriched for GO and KEGG analyses, and the results showed that the significantly associated genes in L96 cotton were enriched for GO, mainly for the organic acid metabolic process, the response to nitrogen compounds, the carboxylic acid metabolic process, and the cellular amino acid metabolic process (Figure 3C). KEGG enrichment was mainly for plant hormone signal transduction; the MAPK signaling pathway—plant, starch and sucrose metabolism, and plant-pathogen interaction (Figure 3D).

### 2.5. Plant Hormone Signal Transduction Pathway Was More Activated

**Figure 4:**
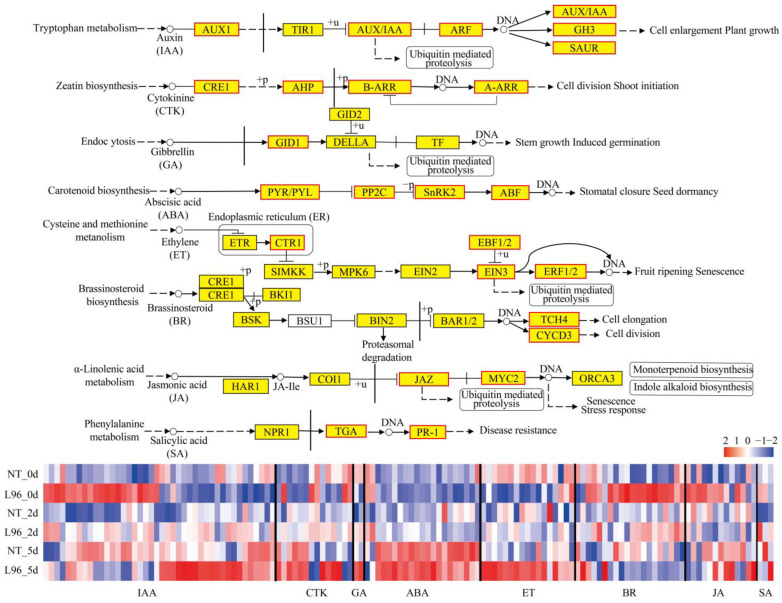
Plant hormone signal pathway activated by L96 and NT under salt stress. Heatmap shows FPKM values in each treatment, normalized using z-score.

After screening using the WGCNA module, L96 cotton was specifically enriched for 130 genes in the plant hormone signaling pathways (Figure 4) (Table S2). Especially after 5 days of salt stress or in the normal growth condition, there were more genes with significantly higher expression in the auxin and cytokinin CTK pathways in L96 compared to NT cotton, which were three *AUX1* genes, thirteen *IAA* genes, four *ARF* genes, six *GH3* genes, and fourteen *SAUR* genes of the auxin (IAA) signaling pathway and eight *CRE1* genes, four *AHP* genes, and one *A-ARR* gene of the CTK signaling pathway. In the gibbrellin (GA) signaling pathway, the expression of two *GID1* genes in L96 cotton was higher than that in NT cotton 2 and 5 days after the salt treatment. In the abscisic acid (ABA) signaling pathway, two *PYL* genes were decreased in L96 cotton compared to NT cotton after 2 days of salt stress, twelve *PP2C* genes were more expressed in L96 cotton than in NT cotton after 5 days of salt stress, one *SnRK1* gene was more expressed in L96 cotton than in NT cotton after 2 and 5 days of salt stress, and six *ABF* genes were more expressed in L96 cotton than in NT cotton after 5 days of salt stress. In the ethylene (ET) signaling pathway, two *CTR1* genes and seven *EBFs* genes were more highly expressed in L96 cotton than in NT cotton after 5 days of salt stress, one *EIN3* gene had lower expression in L96 cotton than in NT cotton in normal growth, one *SnRK1* gene had higher expression in L96 cotton than in NT cotton after 2 and 5 days of salt stress, and six *ERF* genes had higher expression in L96 cotton than in NT cotton after 5 days of salt stress. In the brassinosteroid (BR) signaling pathway, two *BAK1* genes were more highly expressed in L96 cotton than in NT cotton after 5 days of salt stress; one *BIK1* gene was more highly expressed in L96 cotton than in NT cotton after 5 days of salt stress and in normal growth; and two *BSK* genes, one *BZR1/2* gene, and thirteen *TCH4* genes were more highly expressed in L96 cotton than in NT cotton in normal growth. In the jasmonic acid (JA) signaling pathway, ten *JAZ* genes and three *MYC2* genes were more highly expressed in L96 cotton than in NT cotton after 5 days of salt stress or in normal growth conditions. In the salicylic acid (SA) signaling pathway, the two *TGA* genes had lower expression in L96 cotton than in NT cotton during normal growth and salt stress. The *PR* gene was more highly expressed in L96 cotton than in NT cotton after 5 days of salt stress.

### 2.6. Plant–Pathogen Interaction and MAPK Signaling Pathway under Salt Stress

**Figure 5:**
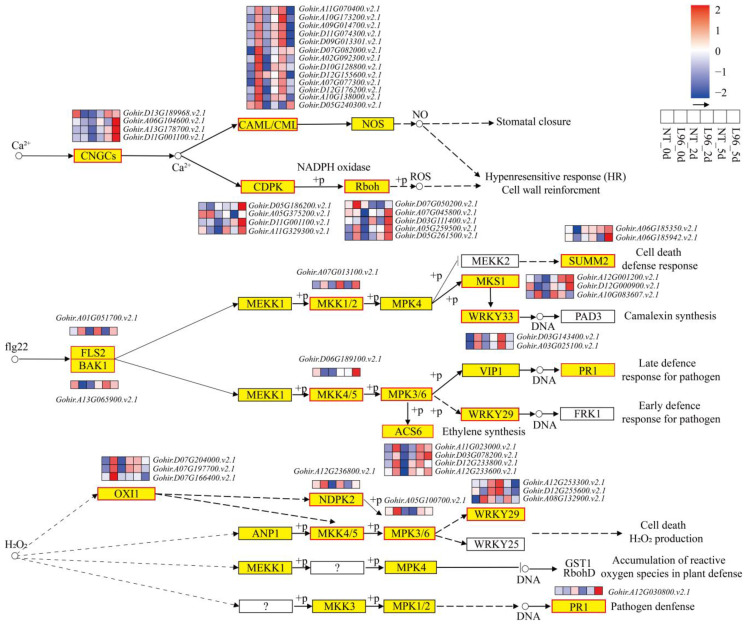
Plant pathogen interaction and MAPK signaling pathway activated by L96 and NT under salt stress. Heatmap shows FPKM values in each treatment, normalized using z-score.

L96 cotton was specifically enriched for 50 genes in the plant-pathogen interaction pathway and the MAPK signaling pathway (Figure 5). In the Ca^2+^ signaling pathway, four *CNGC* genes, four *CDPK* genes, and five *Rboh* genes were expressed in L96 cotton at higher levels than in NT cotton after 5 days of salt stress. Four *CALM* genes and nine *CLM* genes had higher expression in L96 cotton than in NT cotton in normal growth and after 2 days of salt stress but lower expression in L96 cotton than in NT cotton after 5 days of salt stress. In the MAPK signaling pathway, genes mediated by MEKK1-MKK1/2-MPK4-WRKY25/33 and MEKK1-MKK4/5-MPK3/6-WRKY22/29 were specifically expressed in L96 cotton. Two *WRKY33* genes, three *WRKY29* genes, one *NDPK2* gene, and three *OXI1* genes were more highly expressed in L96 cotton than in NT cotton in normal growth and after 2 days of salt stress.

### 2.7. Carbon Fixation in Photosynthetic Organisms Is Benefit of Transgenic Line

**Figure 6:**
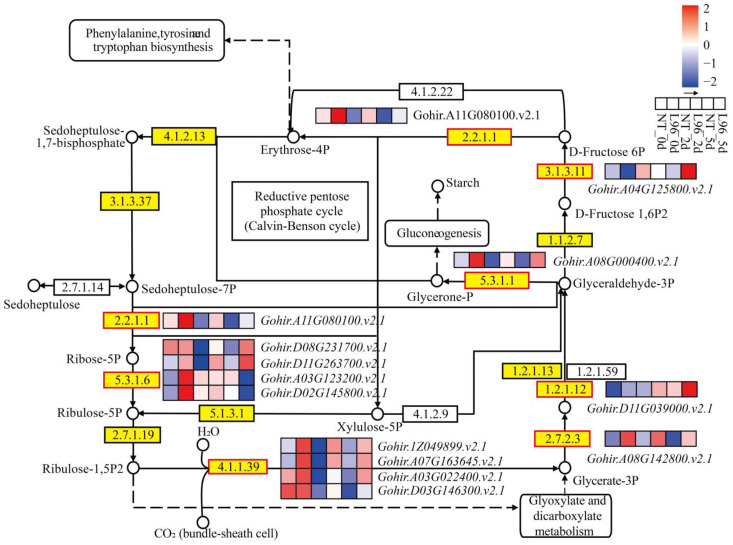
Carbon fixation in photosynthetic organisms activated by L96 and NT under salt stress. Heatmap shows FPKM values in each treatment, normalized using z-score.

L96 cotton was specifically enriched for 25 genes involved in photosynthesis and carbon fixation in photosynthetic organisms (Figure 6). In normal growth, the expression of 10 photosynthesis-related genes (*PsbA*, *PsbB*, *PsbQ*, *PetF*, *PetH*, *atpG*, *atpF*) was higher in L96 cotton than in NT cotton (Figure S4). After 2 days of salt stress, the expression of photosynthesis-related genes were downregulated in NT and L96 cotton, but the expression in L96 cotton was still higher than in NT cotton. After 5 days of salt stress, the expression of photosynthesis-related genes was downregulated to lower levels (Figure S5). Those genes sustained higher levels of photosynthesis proteins, electron transfer chain protein and ATP energy conversion in L96. Moreover, the expression of *rpiA*, *rbcL*, *cbbL*, *tpiA*, *GAPDH,* and other genes involved in carbon fixation was higher in L96 cotton than in NT cotton during normal growth. The expression of these genes was downregulated from 2 days to 5 days of salt stress, but the expression of most genes in L96 cotton was still higher than in NT cotton (Figure 6). These results indicate that assimilation in L96 cotton is better than in NT cotton under either normal or salt stress conditions.

### 2.8. Carbohydrate Metabolism Is Better in Transgenic Line

**Figure 7:**
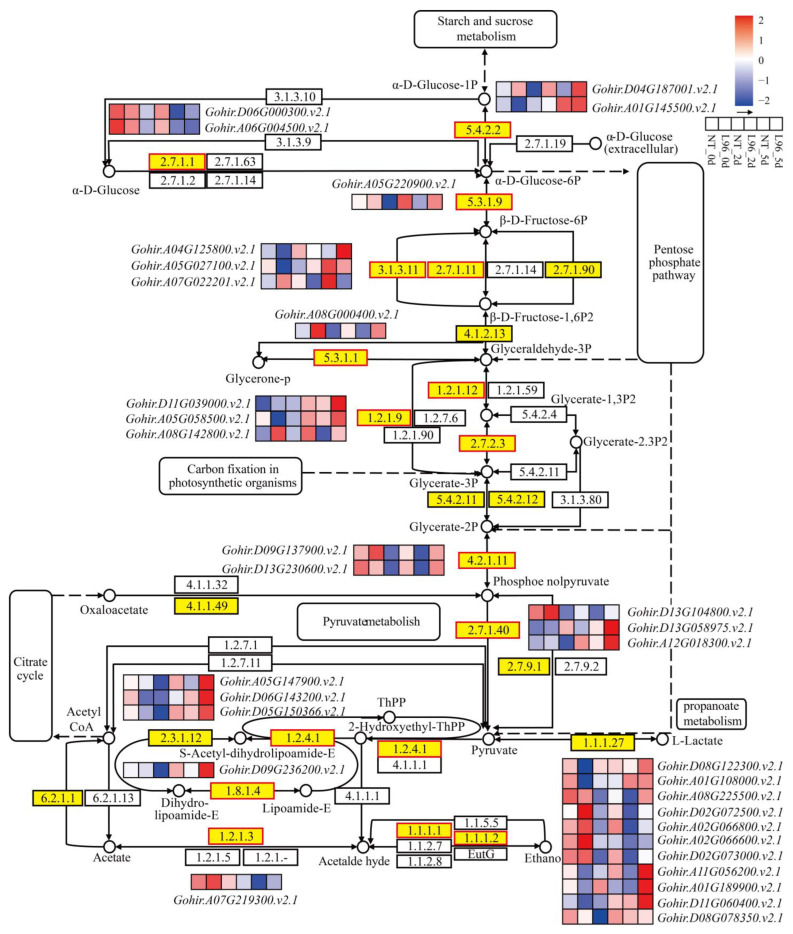
Carbohydrate metabolism activated by L96 and NT under salt stress. Heatmap shows FPKM values in each treatment, normalized using z-score.

In carbohydrate metabolism, 33 genes, including genes involved in glycolysis and the citrate cycle (TCA cycle), were differentially expressed. The expression of genes such as *HK*, *pgm*, *GPI*, *PFK*, *TPI*, *PGK*, *PK*, *ALDH,* and *ADH1* was higher in L96 cotton than in NT cotton during normal growth. The expression of the genes *HK*, *gapN*, *ENO*, *ALDH*, and *ADH1* was higher in L96 cotton than in NT cotton, where the genes *PDHB*, *pyk*, and *pdhD* were expressed after 2 days of salt stress. After 5 days of salt stress, a large number of genes were upregulated, and the expression of *FBP*, *pgm*, *gapN*, *pyk*, *PDHB*, *PDHD*, *ALDH,* and *ADH1* was upregulated and was higher in L96 cotton than in NT cotton (Figure 7). These results indicate that transgenic lines have smoother alienation than NT cotton.

## 3. Discussion

In nature, plants are exposed to a wide range of abiotic stresses. To survive, complex responses are generated within the plant involving multiple morphological, cellular and molecular regulations. *ScALDH21* is a unique gene from a desiccation-tolerant moss, and this gene is found exclusively in this species amongst the moss kingdom [23]. However, overexpression of this gene provided a better phenotype in cotton under salt stress conditions [18,19,20]. Among different environmental factors, salt stress has emerged as a key issue leading to yield losses and significant decreases in crop yield. This research recovered the salt tolerance mechanism of *ScALDH21* transgenic cotton at the physiological and molecular levels. When experiencing salt stress, the transgenic cotton had less damage than the NT cotton, as well as higher PAL activity and proline levels to withstand the ion toxicity and osmotic pressure. For the transcriptomic levels, the transgenic cotton had advantages in transcript factor regulation, plant hormones, signal sensing, photosynthetic assimilation, and carbohydrate metabolism. Furthermore, these molecular regulations balanced the plant growth better under normal and salt stress conditions. This research provides deep insight to support *ScALDH21* transgenic cotton’s salt tolerance ability, which promotes transgenic cotton breeding. At the same time, the results offer some salt stress response genes resources, provide a reference for cotton salt resistance breeding, and provide a reference for the study of the salt tolerance mechanism of cotton.

### 3.1. Effect of Salt Stress on Transcript Levels in Cotton

To survive in a harsh environment, plants regulate their gene expression to quickly respond to stresses [37]. Transcription is the first and most critical step in maintaining the regulation of gene expression [38]. *Arabidopsis thaliana* shows differential gene expression after salt stress, depending on treatment time and the salt concentration, and there is a complex crosstalk between salt stress and other stress signaling pathways [39,40,41]. In the present study, salt stress resulted in the transcriptional reprogramming of a large number of genes in both cotton lines. The number of DEGs between L96 and NT cotton was also highest after 5 days of salt stress. This indicates that the number of genes involved in the transcriptional regulation of cotton increases when plants are continuously exposed to salt stress. Relevant pathways for plants’ response to salt stress that were reported in previous studies were also identified in this transcriptome, such as the plant hormone signaling pathway, the Ca^2+^ signaling pathway, the MAPK signaling pathway, photosynthesis, and carbohydrate metabolism, and a large number of genes in these pathways were transcriptionally reprogrammed [3,42,43], suggesting that these signaling pathways are essential for plants’ responses to abiotic stresses.

Transcriptome analyses of plant salt stress have shown the involvement of all major families of transcription factors, such as bZIP, MYB, NAC, ERF/AP2, and WRKY [44,45]. We screened the key transcription factors involved in salt stress using WGCNA, and the number of *ERF*, *MYB*, *WRKY*, and *NAC* involved in the salt stress process was high (Figure S6). These transcript factors (TF) mainly participate in plant growth and development, and abiotic and biotic stress [45]. Differential TF expression may contribute to the better growth and resistance to salt stress of transgenic cotton. *ScALDH21* is an aldehyde dehydrogenase gene cloned from *S. caninervis*, and its subfamily gene *OsALDH2B1* has been reported to play a regulatory role in the rice growth–defense trade-off [46]. However, it is unknown whether *ScALDH21* can trigger more TF genes in cotton. The *ScAPD1-like* gene from *S. caninervis* was also confirmed to enhance resistance to *Verticillium dahliae* via phenylpropanoid gene regulation [29]. Therefore, we speculated that genes from *S. caninervis*, such as *ScALDH21*, have various functions and still have many excellent genetic resources that we have not tapped.

### 3.2. The Effects of Plant Hormones on the Response of Transgenic Cotton to Salt Stress

Plant hormones are important in the balance between growth and stresses, thereby regulating plant growth and adaptation [47,48]. Auxin is involved in a variety of physiological processes in plants and is a driver of plant development, playing an important role in regulating root and stem structure, leaf morphogenesis, flowering, and senescence [49]. In addition, a large number of auxin-related genes have been shown to enhance salt stress tolerance in plants in previous studies [50]. It was shown in our previous studies that L96 cotton had better growth than NT cotton, which is a reason that the transgene *ScALDH21* might control auxin and its roles in growth and salt tolerance [35,36]. In this study, the expression of auxin-related genes (*AUX*, *IAA* and *ARF*) was higher in L96 cotton than in NT cotton during normal growth, and some other auxin-related genes responded after 5 days of salt stress with higher expression in L96 cotton than in NT cotton. Furthermore, we tested the hormone contents using HPLC-MS. The main auxin IAA contents were higher in the *ScALDH21* transgenic line than in NT cotton 2 and 5 days after the salt treatments and were especially significant on day 0 (Figure S6). We combined the auxin-related DEGs and their contents, and they explained that the transgenic line possessed better growth performance under normal and salt stress conditions. ABA, as one of the most important stress response hormones, plays an irreplaceable role in salt stress defense [51]. In response to salinity and osmotic stress, endogenous ABA levels rapidly increase with enhanced ABA signaling. ABA-related genes expression (*SnRK2*, *PP2C*, and *ABF*) was activated after 5 days of salt stress and was involved in the resistance of cotton to salt stress in L96 cotton, which was similar to NT cotton. However, the content of ABA in L96 cotton was significantly higher than that in NT cotton after salt stress at 0 and 5 days after treatment (Figure S7). We speculated that a higher endogenous ABA content might sustain a higher plant defense balance.

Previous studies have shown that ET accumulates and performs a necessary role in salt stress [51]. *Arabidopsis* treated with ACC exhibited enhanced salt tolerance in all developmental stages, and the mutants *eto1* (ethylene over producer mutant) and *eto2* were also found to confer soil salt tolerance [52]. In this study, the expression of ET-related genes (*ERF* and *EIN*) become upregulated after 2 days of salt stress and further increased after 5 days of salt stress, and the expression of related genes was higher in L96 cotton than in NT cotton. This suggests that ET may also be a key reason for the increased salt tolerance in L96 cotton. Increasing evidence reveals the role of BR in plant adaptation to abiotic stresses [51]. Exogenous BR application alleviated salt-induced growth inhibition in a variety of plants [47,51]. In addition, some key enzymes associated with BR synthesis were found to be required for plant salt adaptation. The upregulation of BR genes to promote GA production can confer salt tolerance in plants [53]. The expression of BR (*TCH4* and *CYCD3*) and GA-related genes (*GID1*) was higher in L96 cotton than in NT cotton during normal growth and salt stress. BR co-regulates salt tolerance in plants by interacting with other plant hormones. JA and SA play an important role in plant salt tolerance in addition to their involvement in plant biotic stresses [47,51]. The importance of JA and SA in the plant response to salt stress signals has been reported in rice, tomato, wheat, and *Arabidopsis* [47,51], and here we also found that these related genes are also induced to be expressed by salt stress. In general, IAA- and BR-related genes had higher expression in the transgenic line than that in NT cotton under the normal growth condition (salt stress day 0). CTK-, IAA-, ET- and ABA-related gene expression was higher in L96 cotton than in NT cotton after salt stress for 5 d. Therefore, *ScALDH21* transgenic cotton has higher growth potential and sustains a higher defense balance level.

### 3.3. The Effects of Ca^2+^ Signaling and the MAPK Pathway on the Response of Transgenic Cotton to Salt Stress

Salt stress leads to an imbalance in cellular ion homeostasis, including triggering an increase in the cytosolic free calcium (Ca^2+^) content ([Ca^2+^] cyt) [54]. It was found that locally restricted increases in Ca^2+^ in roots in response to salinity stress subsequently triggered a systemic molecular response in a whole plant within seconds, suggesting an effective role of Ca^2+^ signaling in salinity tolerance [54]. The expression of *CML* and *CALM* genes was higher in L96 cotton on days 0 and 2 after salt stress treatment, but in NT cotton the expression was higher after 5 days of salt stress. *CDPK* expression was higher in L96 cotton than in NT cotton after 5 days of salt stress treatment. These results suggest that L96 pairs may enhance resistance by sensing early cell signaling more quickly, although it is not clear why. This may be one of the reasons why L96 cotton is tolerant to salt stress. ROS are important signaling molecules for plants in response to abiotic stress [11]. *Rboh* expression was higher than NT cotton during both normal growth and salt stress, especially after salt stress for 5 days. It was shown that salt-induced ABA and Ca^2+^ signals activate *RBOHF* activity through *SnRK2.6* and *CIPK11/26* signaling modules [47,51]. Thus, the signaling crosstalk of ABA, ROS, and Ca^2+^ is a key in controlling the plant response to salt stress. The MAPK cascade reaction, as one of the most important signal transduction pathways in plants, can gradually amplify environmental signals through protein phosphorylation catalyzed by protein kinases [43]. In the present study, MAPK-related genes were induced to be expressed after salt stress in cotton and led to downstream gene responses (*SUMM2*, *MKS1*, and *WRKY29/33*). The expression of *WRKY29/33* was higher in L96 cotton on days 0 and 2 of salt stress, but the expression was higher in NT cotton after 5 days of salt stress. It was shown that salt stress not only activates *ACS7* at the transcriptional level but also phosphorylates and stabilizes *ACS2/6* at the protein level by activating the MAPK signaling response. This is consistent with data that found that the expression of *ACS6* was similarly elevated in salt stress [55]. Both the MAPK signaling pathway and the Ca^2+^ signaling pathway are able to respond earlier, and we therefore speculate that this is essential for the resistance of L96 cotton to salt stress.

### 3.4. The Effects of Photosynthesis on the Response of Transgenic Cotton to Salt Stress

Photosynthesis is the most important process occurring in the chloroplasts of higher plants, and for many plants, salt stress affects the chloroplast structure, decreases the chlorophyll content, and leads to a decrease in photosynthetic rate [56]. In this study, we found that the expression of photosynthesis-related genes was higher in L96 cotton than in NT cotton, which is consistent with previous studies and may be responsible for the higher growth in L96 cotton compared to NT cotton [35,36]. Previous studies showed that the net photosynthetic rates and the chlorophyll contents of transgenic plants in the flowering stage under different irrigation treatments were higher than those of NT plants [32]. In addition, during salt stress, photosynthesis-related genes were downregulated in both L96 and NT cotton, but the expression of L96 cotton was always higher than that of NT cotton. For carbon fixation in photosynthetic organisms, the expression of most genes was equally higher in L96 cotton compared to NT cotton during normal growth and salt stress. These results are similar to those of many plants in response to salt stress and suggest that photosynthesis plays a role in the resistance of L96 cotton to salt stress and growth.

### 3.5. The Effects of Carbohydrate Metabolism on the Response of Transgenic Cotton to Salt Stress

The carbohydrate and energy metabolism in many plants is severely affected by salt stress [57]. In this study, most of the DEGs were involved in carbohydrate and energy metabolism, including glycolysis/gluconeogenesis, the citric acid cycle, and carbon fixation. Interestingly, *GAPDH* an important and highly conserved glycolysis-related gene in the glycolytic/glycoisogenic pathway, was more highly expressed in L96 cotton than in NT cotton in both normal growth and salt-stressed conditions. *GAPDH* was shown to mediate ROS signaling and contribute to the maintenance of cellular ATP levels, carbohydrate metabolism, and normal fertility in *Arabidopsis*. Most plant *GAPDH* genes have been shown to be induced under various stresses, and the overexpression of *GAPDH* genes also helps to alleviate plant responses to stresses [58,59]. In addition, some genes such as *HK*, *PGM*, *GPI*, *PFK*, *TPI*, *PGK*, *PK*, *ALDH*, and *ADH1* were induced in plants in response to stresses such as drought, salt, and flooding. In general, the increased expression of these genes in plants after salt treatment may be related to salt tolerance. Further studies should investigate the molecular functions of these genes to better understand the mechanisms involved in the salt tolerance of L96 cotton.

## 4. Materials and Methods

### 4.1. Plant Material and Cultured Conditions in Greenhouse

To discover the mechanism of salt tolerance, the *ScALDH21* transgenic line (L96) carrying the heterologous *ScALDH21* gene of *Syntrichia caninervis* moss and its recipient non-transgenic (NT) cotton cultivar Xinnongmian 1 line were employed to analyze their responses on the physiological and transcriptional levels under salt stress conditions. The transgenic L96 cotton line was obtained in our previous research [30,35]. Both lines were grown in a greenhouse. The soil substrate was prepared by mixing black hill soil, charcoal soil, vermiculite, and perlite in a 5:3:1:1 ratio and autoclaved at 121 °C for 25 min. Further, the soil substrate was moistened with water to reach 60% moisture. The moistened soil mixture was placed in a small square box and gently compacted by hand, and 18 boxes were placed in a water storage tray. The cotton seeds were depilated using concentrated sulfuric acid and washed with water. The germinating seeds were selected, soaked in sterile water and placed in a constant temperature incubator at 30 °C overnight. Five seeds were sown with the root tips facing down in each box (one in each corner and the center), slightly pushed into the soil substrate, and covered with the soil. Irrigation was performed 2–3 times per week, and salt stress (150 mM NaCl) was applied when they grew to 1 month old.

### 4.2. Measurement of Physiological Indicators

The physiological indicators: PAL, H_2_O_2_, MDA, Pro, and POD were measured using detection assay kits (Cat. No. A137-1-1, A064-1-1, A003-3-1, A107-1-1 and A084-3-1 respectively; Nanjing Jiancheng Bioengineering Institute, China). All the tests were performed according to the manufacturer’s instructions (http://www.njjcbio.com/, accessed on 1 December 2022). A lignin test kit was obtained from COMIN company (Cat. No. MZS-1-G; Suzhou, China). The leaves were used for physiological detection. Expanded leaves were collected from L96 and NT plants after salt stress (0 d, 2 d and 5 d). Each sample was repeated 4 times. At the same time, root were collected, washed, and frozen in −80 °C for the following RNA extraction and plant hormone detection via HPLC (Agilent 1260, Santa Clara, CA, USA).

### 4.3. RNA Extraction and Quantitative PCR

Total RNA was extracted from the roots using TRIzol^®^ Reagent (Plant RNA Purification Reagent for plant tissue) according to the manufacturer’s instructions (Invitrogen), and genomic DNA was removed using DNase I (TaKara, Beijing, China). RNA degradation and contamination were monitored on 1% agarose gels. Then, the RNA quality was determined using a 2100 Bioanalyser (Agilent Technologies, Santa Clara, CA, USA) and quantified using an ND-2000 (Nano Drop Technologies, LLC, located in Wilmington, DE, USA). Only high-quality RNA samples were used to construct a sequencing library. Next, 18S rRNA were used as reference genes for normalization. Relative abundance levels were calculated using the 2^−ΔΔCt^ method with three biological replicates and three technical replicates. The primers used for real-time fluorescence quantitative PCR experiments were designed using the NCBI primer BLAST program and are listed in Supplementary (Table S1).

### 4.4. cDNA Library Preparation and Sequencing

RNA purification, reverse transcription, library construction, and sequencing were performed at Shanghai Majorbio Bio-pharm Biotechnology Co., Ltd. (Shanghai, China) according to the manufacturer’s instructions (Illumina, San Diego, CA, USA). The transcriptome library was prepared using a TruSeqTM RNA sample preparation kit from Illumina (San Diego, CA, USA) using 1 μg of total RNA. Briefly, messenger RNA was first isolated according to the polyA selection method using oligo (dT) beads and then fragmented using a fragmentation buffer. Second, double-stranded cDNA was synthesized using a SuperScript double-stranded cDNA synthesis kit (Invitrogen, CA, USA) with random hexamer primers (Illumina). Then, the synthesized cDNA was subjected to end-repair, phosphorylation, and ‘A’ base addition according to Illumina’s library construction protocol. Libraries were size selected for 300 bp cDNA target fragments on 2% low range ultra agarose (Biofroxx, GER), followed by PCR amplified using Phusion DNA polymerase (New England Biolabs, Beijing, China ) for 15 PCR cycles. After quantification using TBS380 (YPH-Bio, Beijing, China), a paired-end RNA-seq sequencing library was sequenced using an Illumina NovaSeq 6000 sequencer (2 × 150 bp read length).

### 4.5. Quality Control and Read Mapping

The raw paired-end reads were trimmed and quality-controlled using fastp (https://github.com/OpenGene/fastp, accessed on 1 November 2022) with default parameters. Then, clean reads were separately aligned to the reference genome using HISAT2 (http://ccb.jhu.edu/software/hisat2/index.shtml, accessed on 1 November 2022) orientation modeling software. The mapped reads of each sample were assembled using StringTie (https://ccb.jhu.edu/software/stringtie/, accessed on 1 November 2022) in a reference-based approach. The reference genome was *Gossypium_hirsutum* (Ghirsutum_527, https://phytozome-next.jgi.doe.gov/info/Ghirsutum_v2_1, accessed on 1 November 2022).

### 4.6. Differential Expression Analysis and Functional Enrichment

To identify DEGs (differentially expressed genes) between two different samples/groups, the expression level of each gene was calculated according to the transcripts per million reads (TPM) method. RSEM (http://deweylab.biostat.wisc.edu/rsem/, accessed on 1 December 2022) was used to quantify gene abundances. Essentially, a differential expression analysis was performed using DESeq2, DEGseq edgeR, Limma, NOIseq, and DEGs with log_2_(fold change) ≥ 1 and *p*-adjust ≤ 0.05 [60]. Functional enrichment analyses, including GO (Gene Ontology, http://www.geneontology.org, accessed on 1 December 2022) and KEGG (Kyoto Encyclopedia of Genes and Genomes, http://www.genome.jp/kegg/, accessed on 1 December 2022), were performed to identify which DEGs were significantly enriched in GO terms and metabolic pathways at *p*-adjust ≤ 0.05 compared with the whole-transcriptome background. GO functional enrichment, KEGG pathway analysis, and WGCNA (weighted correlation network analysis)were conducted using the Majorbio platform [60].

### 4.7. Statistical Analysis

Statistical analyses were performed using SPSS 20 (IBM, Armonk, NY, USA) software. All data consisted of the means of at least three independent replicates. A one-way ANOVA with Tukey tests was used to determine the statistical significance; ns indicates no significant difference. *, **, and *** specify statistical significance at *p* < 0.05, *p* < 0.01, and *p* < 0.001, respectively. The column graphs were generated using GraphPad Prism 9 (Version 9.0.0, 2020) software.

## 5. Conclusions

Presently, transgenic breeding is attracting more and more attention. In this study, we discovered the salt resistant mechanism of *ScALDH21* transgenic cotton based on physiological and transcriptome profiles. Transgenic *ScALDH21* cotton showed a better salt tolerance phenotype, mainly through (1) increases in the PAL and PRO levels to resist salinity and osmotic stress; (2) decreases in theMDA and H_2_O_2_ levels to avoid cell damage; (3) regulated hormone-related gene expression to sustain higher ABA and IAA contents to balance resistance and growth; (4) a stress signal response that was more sensitive; and (5) smoother photosynthesis carbon fixation and carbohydrate metabolism. Therefore, we report that the heterogenous gene *ScALDH21* from an extremely drought-tolerant moss triggered a salt tolerance molecular pathway, induced physiological combat in cotton, and provided a better-performing phenotype under salt stress conditions. This research offers a new cotton breeding strategy and some insights into the mechanism of salt tolerance in cotton.

## Data Availability

The data supporting this study’s findings have been deposited into the CNGB Sequence Archive (CNSA) of the China National GeneBank DataBase (CNGBdb) with accession numbers CNP0003933 (https://db.cngb.org/, accessed on 13 January 2023).

## Supplementary Materials

The supporting information can be downloaded at: https://www.mdpi.com/article/10.3390/ijms24065822/s1.
