# Supplementary material for: Transcriptome Reveals the Molecular Mechanism of the ScALDH21 Gene from the Desert Moss Syntrichia caninervis Conferring Resistance to Salt Stress in Cotton
Source: Int J Mol Sci. 2023 Mar 18;24(6):5822. doi: 10.3390/ijms24065822 (PMC10053822; doi:10.3390/ijms24065822)
Supplement: Supplementary file 1 [file ijms-24-05822-s001.zip › ijms-2256100 supplementary.pdf]

**Supplementary Data:**

**Table S1. Real-time fluorescence quantitative PCR primer sequence information.**

| Primer name | Sequence (5′-3′) | Tm [°C) | GC (%) |
|---|---|---|---|
| Gohir.D07G214800.v2.1-F | CGTGCGTGGGGCTCTATTAT | 59.97 | 55.00 |
| Gohir.D07G214800.v2.1-R | CGGTATGATCGGACTGGTGG | 59.97 | 60.00 |
| Gohir.A04G039105.v2.1-F | CGACTAGTTCCGGGTTCGAG | 59.90 | 60.00 |
| Gohir.A04G039105.v2.1-R | CCATAGGCTTTCGCTTTCGC | 59.97 | 55.00 |
| Gohir.D13G003200.v2.1-F | CACTTGGCAGGGCATCTAA | 60.03 | 55.00 |
| Gohir.D13G003200.v2.1-R | CCGTTTCTTGGGGTCTCGAA | 59.97 | 55.00 |
| Gohir.D12G233800.1.v2.1-F | GGCCAGATCCACCATAGCTC | 59.96 | 60.00 |
| Gohir.D12G233800.1.v2.1-R | CACCGCTCATAACAATGCGG | 59.97 | 55.00 |
| 18S rRNA-F | CGGCTACCACATCCAAGGAA | 59.75 | 55.00 |
| 18S rRNA-R | TGTCACTACCTCCCCGTGTCA | 62.56 | 57.14 |

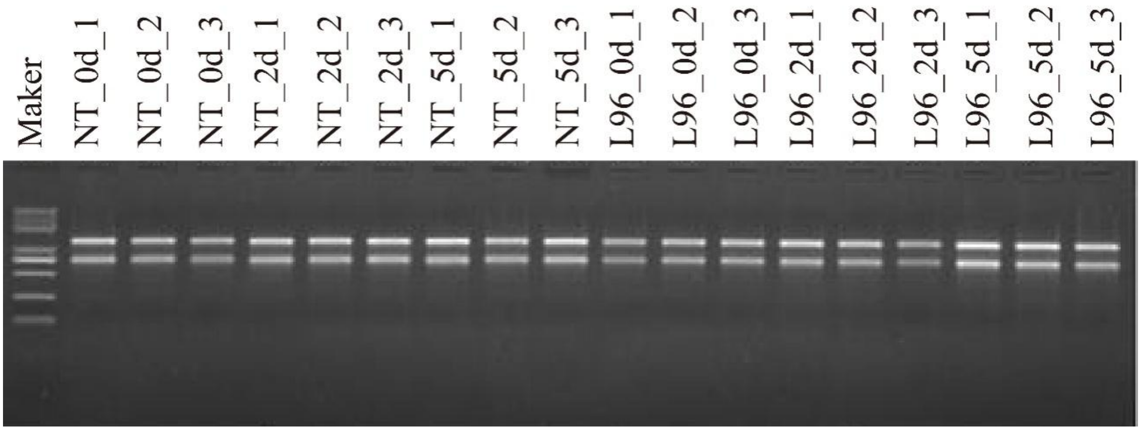


**Figure S1.** Transcriptome sequencing RNA integrity assay.

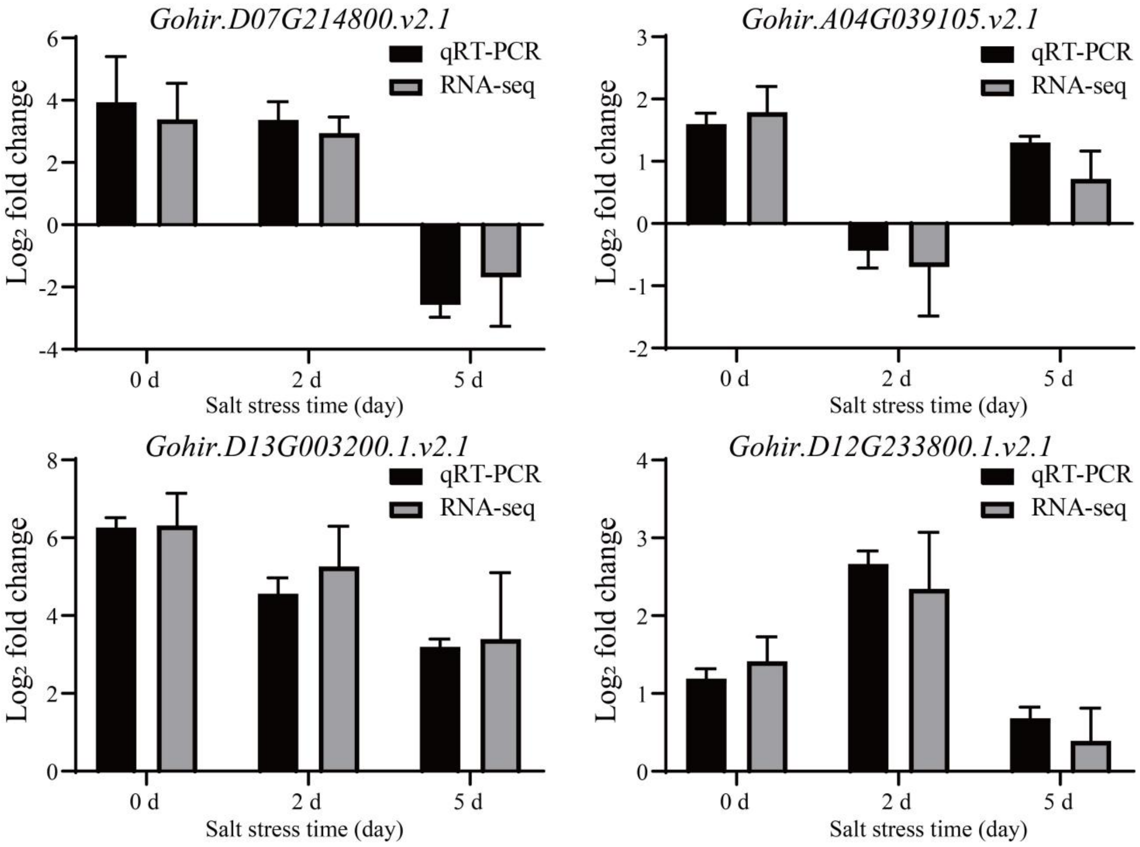


**Figure S2.** Validation of NT and L96 transcriptome under salt stress. The transcriptome data was $\log_2$ fold change using FPKM, real-time fluorescence quantitative PCR gene expression data were shown as the mean ± SD, n=3.

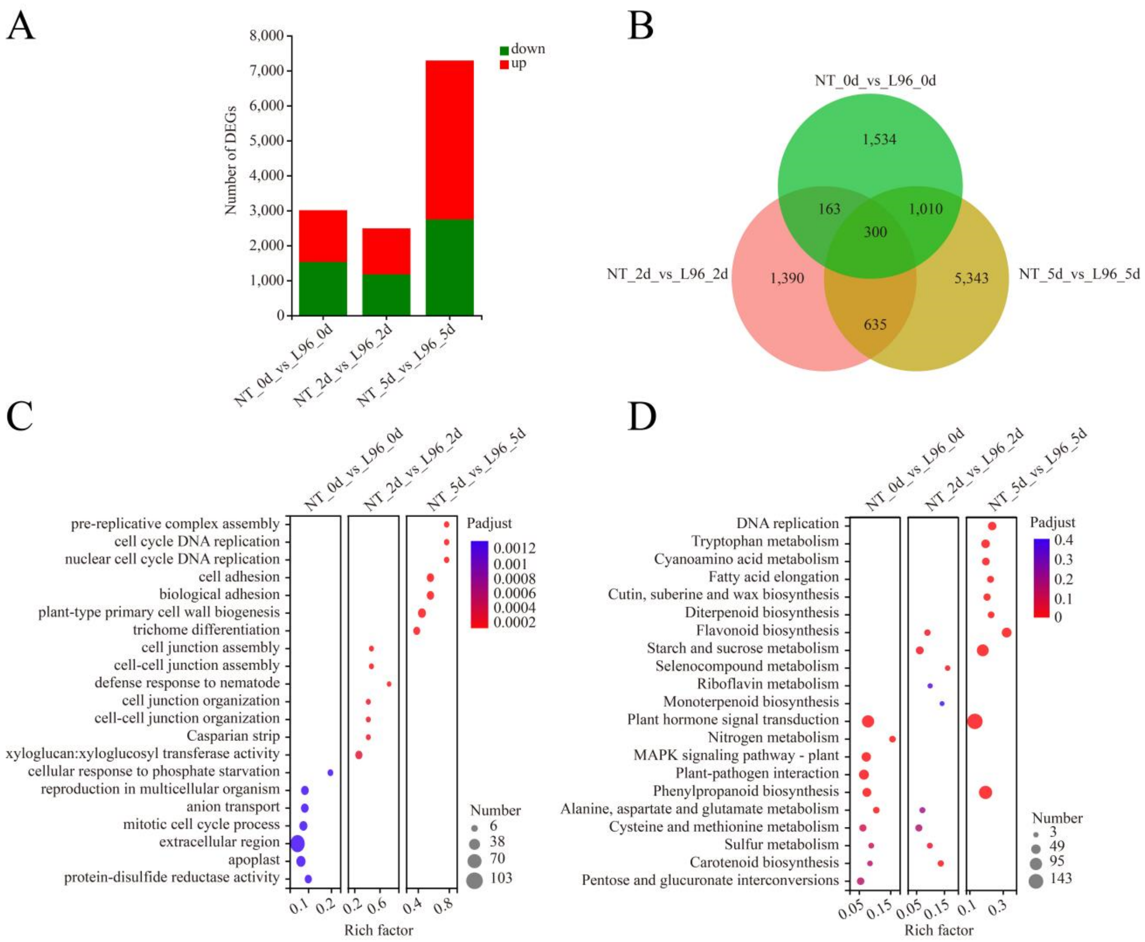


**Figure S3.** NT and L96 differential gene enrichment analysis. (**A**) Differential expression analysis of samples under salt stress. (**B**) Venn analysis of DEGs. (**C**) GO enrichment analysis of DEGs. (**D**) KEGG enrichment analysis of DEGs.

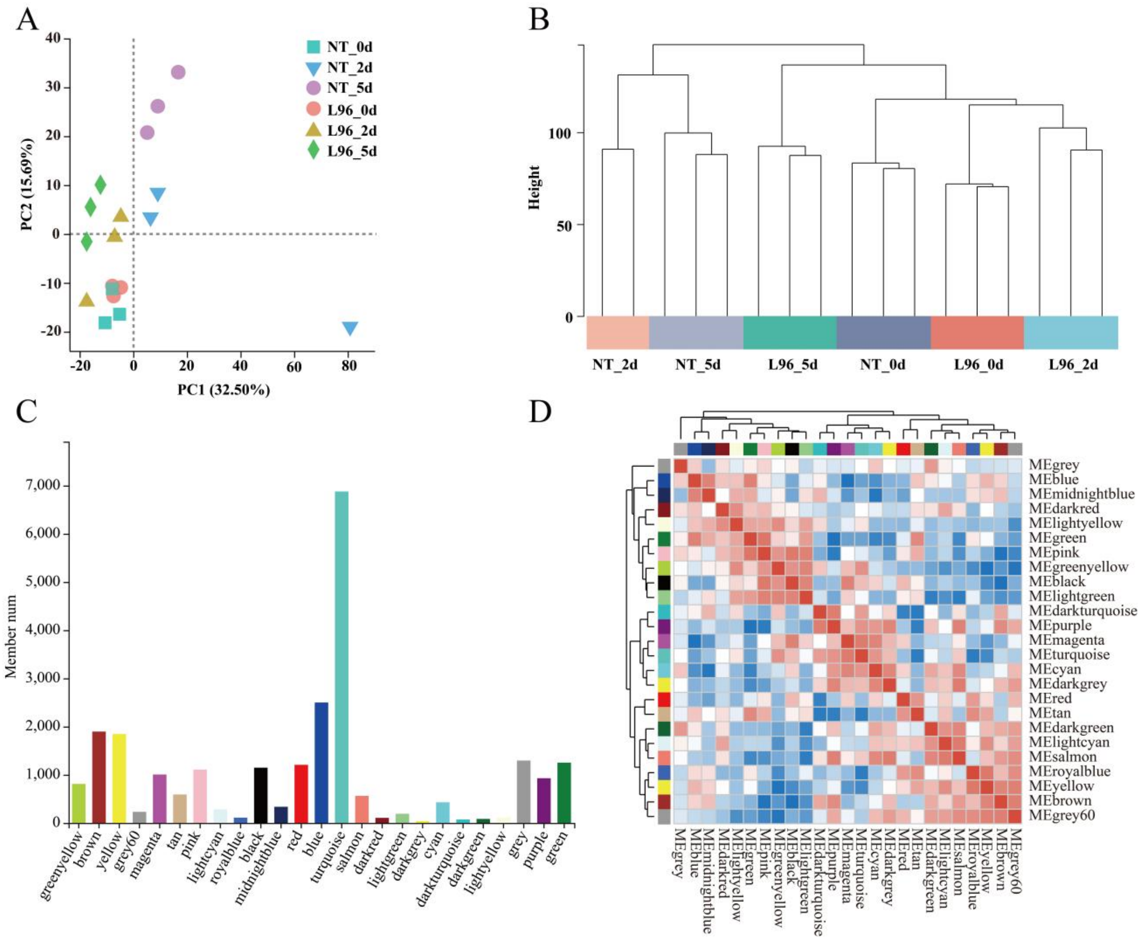


**Figure S4.** NT and L96 salt stress transcriptome WGCNA analysis. (**A**) Sample PCA analysis. (**B**) Sample clustering analysis. (**C**) WGCNA module identification. (**D**) WGCNA module correlation analysis.

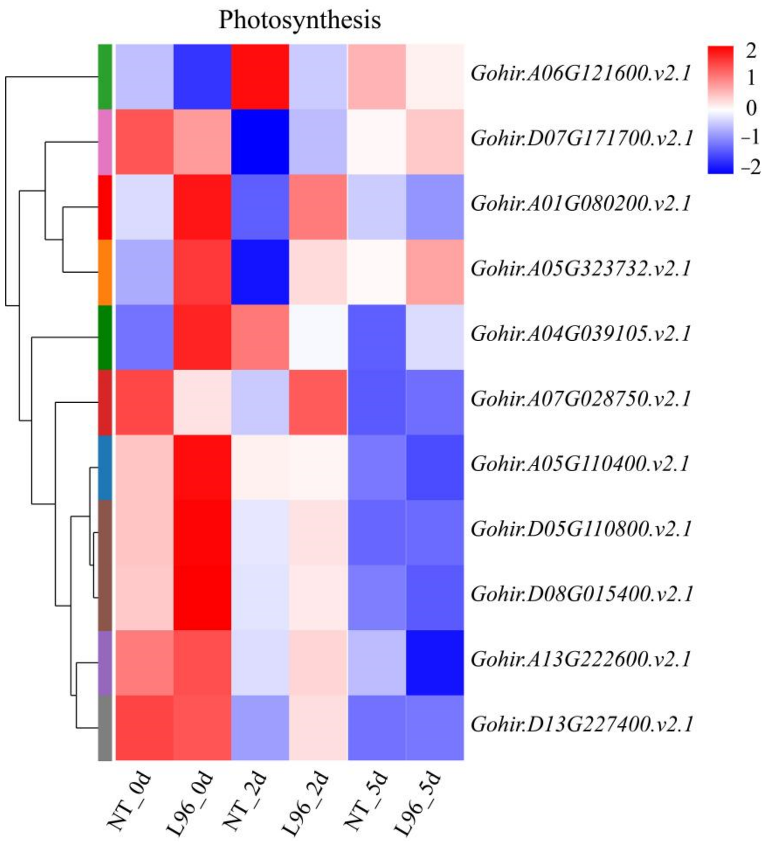


**Figure S5.** photosynthesis activated by L96 and NT under salt stress. Heatmap shows FPKM values in each treatment, normalized using z-score.

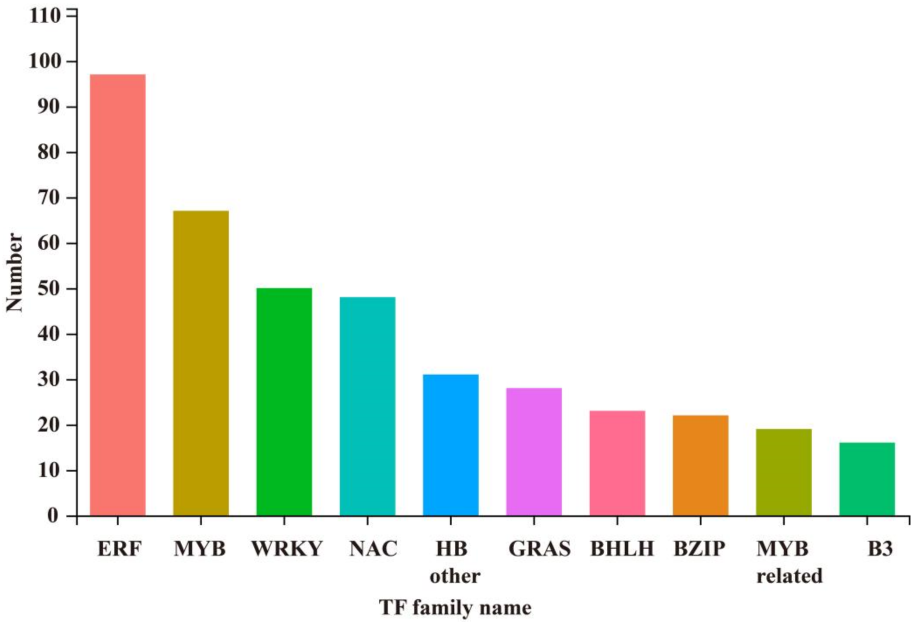


**Figure S6.** Statistics on the number of transcription factor families in WGCNA analysis.

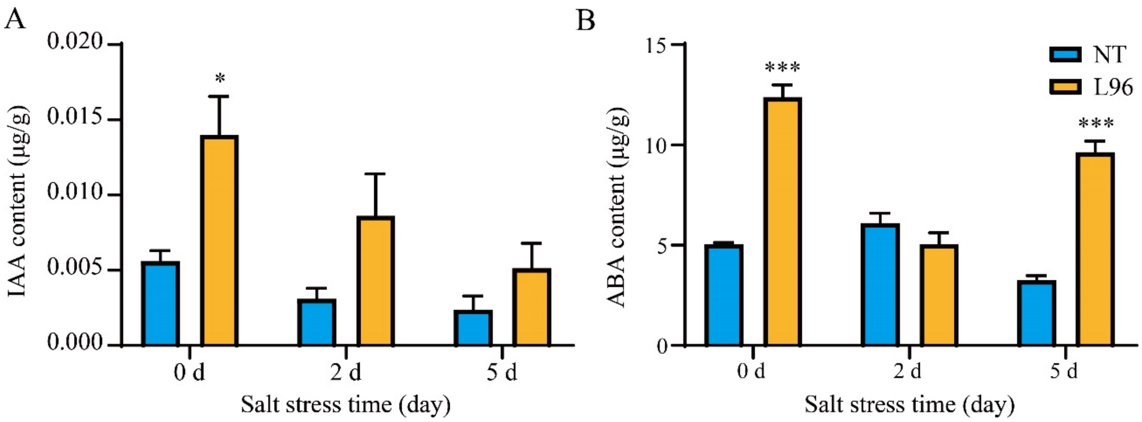


**Figure S7.** IAA and ABA content of NT and L96 plant under salt stress. Data represent the mean ± SD from three biological replicates. Significance was determined by the least significant difference, asterisks indicate statistically significant differences from NT (*$P < 0.05$, **$P < 0.01$, ***$P < 0.001$).
